# Comparison of the pathogenesis of the highly passaged MCMV Smith strain with that of the low passaged MCMV HaNa1 isolate in BALB/c mice upon oronasal inoculation

**DOI:** 10.1186/s13567-015-0228-6

**Published:** 2015-09-04

**Authors:** Shunchuan Zhang, Jun Xiang, Jan Van Doorsselaere, Hans J. Nauwynck

**Affiliations:** Laboratory of Virology, Department of Virology, Parasitology, and Immunology, Faculty of Veterinary Medicine, Ghent University, Merelbeke, Belgium; Higher Institute for Nursing and Biotechnology, VIVES University College, Roeselare, Belgium

## Abstract

Murine cytomegalovirus (MCMV) Smith strain is widely used in mouse models to study HCMV infections. Due to high serial passages, MCMV Smith has acquired genetic and biological changes. Therefore, a low passaged strain would be more relevant to develop mouse models. Here, the pathogenesis of an infection with MCMV Smith was compared with that of an infection with a low passaged Belgian MCMV isolate HaNa1 in BALB/c adult mice following oronasal inoculation with either a low (10^4^ TCID_50_/mouse) or high (10^6^ TCID_50_/mouse) inoculation dose. Both strains were mainly replicating in nasal mucosa and submandibular glands for one to two months. In nasal mucosa, MCMV was detected earlier and longer (1–49 days post inoculation (dpi)) and reached higher titers with the high inoculation dose compared to the low inoculation dose (14–35 dpi). In submandibular glands, a similar finding was observed (high dose: 7–49 dpi; low dose: 14–42 dpi). In lungs, both strains showed a restricted replication. In spleen, liver and kidneys, only the Smith strain established a productive infection. The infected cells were identified as olfactory neurons and sustentacular cells in olfactory epithelium, macrophages and dendritic cells in NALT, acinar cells in submandibular glands, and macrophages and epithelial cells in lungs for both strains. Antibody analysis demonstrated for both strains that IgG_2a_ was the main detectable antibody subclass. Overall, our results show that significant phenotypic differences exist between the two strains. MCMV HaNa1 has been shown to be interesting for use in mouse models in order to get better insights for HCMV infections in immunocompetent humans.

## Introduction

Human cytomegalovirus (HCMV), also known as human herpesvirus 5 (HHV-5), is the prototype member of the *Betaherpesvirinae* within the family of the *Herpesviridae*. It is an ubiquitous virus with a worldwide distribution [1]. It is the main cause of congenital infections in the world, affecting 0.5–2.0% of live births [2,3] and leading to central nervous damage, congenital deafness and mental retardation [4-7]. HCMV infection is also harmful for the immunocompromised individuals, such as HIV patients and recipients of organ transplants, but is in general asymptomatic in the immunocompetent host [8].

Due to the strict species-specificity of HCMV, it is not possible to study this virus in experimental animals. Therefore, it is necessary to set up animal models for the study of HCMV. Up till now, a number of CMV infections in various animal species have been utilized for modeling HCMV infection. Among the animal CMVs, pathogenesis has been reported for rhesus macaque CMV, porcine CMV, guinea pig CMV, rat CMV and murine CMV [9-13]. The mouse model with MCMV is the most commonly and widely used animal model for HCMV study, due to the following reasons: (1) MCMV shares many features with HCMV [4], (2) the genomes of mice and MCMV are fully sequenced [14,15] and (3) the small size, short life span, ease of handling and high reproductive rate make them most suitable.

MCMV has been studied for more than 60 years. Most published studies used the MCMV Smith strain or MCMV K181 derived from the Smith strain, which were highly passaged in vitro or in vivo. It is now apparent that strains or variants of MCMV Smith that are in common use have acquired genetic and biological differences during passaging [16,17]. The same problem has been discussed for HCMV, where serially passaged laboratory strains, such as the commonly used HCMV AD169, exhibit significant biological differences compared to the clinical isolates of HCMV [18,19]. Therefore, using serially passaged strains of MCMV may not be able to reproduce the full range or extent of virus replication and clinical outcome that are associated with HCMV infections. It is important that more emphasis is being placed on the use of recent isolates of MCMV and avoiding cell culture passaging of these isolates.

Besides the passage history, the inoculation route is an important factor during in vivo studies as well. The inoculation route should mimic the natural route of MCMV infection. Most of the published studies on MCMV have utilized the intraperitoneal inoculation, sometimes intracerebral, orbital or intravenous inoculation, none of which can be considered as being natural [20]. Although intramuscular or subcutaneous inoculation mimics natural infection upon biting, only intranasal and oral inoculations are widely accepted as the route of natural infection. Unfortunately, there is very limited information on natural infection upon oronasal inoculation, with only a few studies on viral kinetics, organ and tissue tropism, and host response [21-23].

In the present study, we have used two MCMV strains (low passaged MCMV HaNa1 isolate and highly passaged MCMV Smith strain) to set up mouse models using the natural route of infection (oronasally) with a low (10^4^TCID_50_ per mouse) and high (10^6^TCID_50_ per mouse) inoculation dose without sedation/anesthesia in order to compare the pathogenesis of a low passaged isolate HaNa1 and the well-studied Smith strain.

## Materials and methods

### Ethics statement

All animal experiments (Case number 2013–47) were approved by the local Ethical Committee of the Faculty of Veterinary Medicine, Ghent University.

### Cells and viruses

Primary BALB/c mouse whole fetus cells (MWFc) at passage 2 were propagated at 37 °C and 5% CO_2,_ in minimum essential medium with 10% fetal calf serum (FCS) and a mixture of antibiotics (100 U/mL penicillin, 100 μg/mL streptomycin and 50 μg/mL gentamicin). Viruses used in the present experiments were the second passage of clone1 of the MCMV HaNa1 isolate, which was isolated by our laboratory from a domestic mouse, and the MCMV Smith strain at unknown passage. Up till now, seven parts of the MCMV HaNa1 genome have been sequenced and submitted to GenBank: m06 gene (accession No.: KR184668), m033 gene (accession No.: KR184669), mck-2 including exon1 (m131 gene) and exon2 (m129 gene) (accession No.: KR184670), m138 gene (accession No.: KR184671), m144 gene (accession No.: KR184672), m152 gene (accession No.: KR184673) and m157 gene (submission ID: 1840944).

### Multistep growth curves of two MCMV strains in MWFc

In order to have a better understanding of the in vitro viral replication kinetics of both MCMV strains, a growth curve analysis was performed. Monolayers of MWFc in 24-well plates were inoculated in triplicate with MCMV HaNa1 or MCMV Smith at 10^4^ TCID_50_/well. After inoculation for 1 h at 37 °C with 5% CO_2,_ the inoculum was removed, and cells were washed three times with 2 mL PBS. Afterwards, 1 mL of fresh culture medium was added per well. The supernatants (1 mL) with the extracellular virus and the infected cells containing intracellular virus, which were resuspended in 1 mL PBS, were collected at 1, 12, 24, 48 and 72 hpi. The virus inactivation curve was determined by keeping cell free virus in culture medium at 37 °C with 5% CO_2_. Samples were taken at different time points. The samples were stored at −70 °C upon use at the end of the experiment. All samples were thawed and cleared of cellular debris, and then titrated to determine 50% tissue culture infectious dose (TCID_50_) according to the Reed and Muench formula [24].

### Animals and virus inoculation

A total of 135 specific pathogen-free 8-week-old BALB/c female mice were used. In both low dose groups (36 mice/group), each mouse was inoculated with 100 μL PBS containing 10^4^ TCID_50_ MCMV HaNa1 or MCMV Smith via intranasal (25 μL) and peroral (75 μL) routes without sedation/anesthesia. For the intranasal inoculation, a small amount of inoculum (5 μL) was repeatedly instilled in each nostril. Each application was done with several minutes interval. For the oral inoculation, 25 μL inoculum was given three times with a few minutes interval between each inoculation. Mice were kept in isolation and fed *ad libitum*. Three inoculated mice were euthanized at each time point (1, 3, 5, 7, 10, 14, 17, 21, 28, 35, 42 and 49 days post inoculation (dpi)). In both high dose groups (30 mice/group), each mouse was inoculated with 100 μL PBS containing 10^6^ TCID_50_ MCMV HaNa1 or MCMV Smith via intranasal (25 μL) and peroral (75 μL) routes using the same methodology. Three infected mice were euthanized at each time point (1, 3, 5, 7, 10, 14, 17, 21, 35 and 49 dpi). Another 3 mice were mock inoculated with PBS and euthanized at the end of the experiment.

### Collection of saliva, blood and tissues

Saliva was collected by swabs and stored in 0.3 mL of cold sterile PBS containing 1% fetal calf serum and a mixture of antibiotics (100 U/mL penicillin, 100 μg/mL streptomycin and 50 μg/mL gentamicin). Upon anesthesia with 130 μL of 10 mg/mL sodium pentobarbital (KELA, Belgium) per mouse, 0.5 mL blood was collected from the orbital sinus with a heparinized pasteur pipet and kept in an eppendorf with 0.5 mL PBS containing 5 U/mL heparin (Leo Pharma, Zaventem, Belgium). Then, plasma was harvested through centrifugation (200 *g* for 10 min) and stored at −70 °C for virus and antibody titration. Peripheral blood mononuclear cells (PBMC) were isolated on a Ficoll-Paque cushion according to manufacturer’s protocol (GE Healthcare), washed three times, resuspended in 0.5 mL RPMI and counted with a haemocytometer. The fresh PBMC were used for co-culture studies. After blood collection, mouse was euthanized with 200 μL of 10 mg/mL sodium pentobarbital (KELA, Belgium). Various tissues were collected under aseptic conditions from the nerve system (olfactory bulb and brain), from the respiratory system (nasal mucosa, nasopharynx-associated lymphoid tissues (NALT), pharynx, trachea and lungs), from the alimentary system (submandibular glands, esophagus and small intestines), from the abdominal organs (liver and kidneys), from the reproductive system (uterus and ovaries) and from the lymphoid organs (thymus and spleen). One part of an organ was stored at −70 °C for virus titration. The other part was snap frozen with methocel and stored at −70 °C for immunofluorescence staining.

### Virus titration of tissues

A five percent homogenate was made of all collected tissues for virus titration. Briefly, tissues were thawed, weighed and homogenized by using a pestle, a small volume of sterile sand and DPBS with 0.9 mM CaCl_2_, 0.5 mM MgCl_2_ × 6H_2_O and 0.002% phenol red, supplemented with 2% FCS and a mixture of antibiotics (100 U/mL penicillin, 100 μg/mL streptomycin and 50 μg/mL gentamycin). Afterwards, the supernatants were collected after centrifugation (2400 *g*, 10 min). Virus titration was performed on the second passage of MWFc. After 7 days, the presence of a cytopathic effect (CPE) was assessed by light microscopy (Olympus Optical Co., Hamburg, Germany) and virus titer was calculated as 50% tissue culture infectious dose (TCID_50_) according to the Reed and Muench formula [24].

### Co-culture of PBMC

To examine the cell-associated viremia in PBMC, co-culture assays were performed. MWFc (2 × 10^5^/well) were seeded in 24-well plates two days prior to co-culture. Freshly isolated PBMC were brought on the monolayer (PBMC from one mouse were equally divided into 2 wells of a 24-well plate) and covered with 1 mL carboxymethylcellulose (CMC) medium (1/4 2xMEM, 1/4 2 × RPMI, 1/2 2 × CMC supplemented with 5% FCS, 100 U/mL penicillin, 100 μg/mL streptomycin, 50 μg/mL gentamicin, 0.1 mM non-essential amino acids (NEAA) and 1 mM Sodium pyruvate), and the plates were centrifuged 750 g for 10 min, afterwards cultivated at 37 °C in the incubator for 8 days. Plaques were counted with a light microscopy (Olympus Optical Co., Hamburg, Germany).

### Production of biotinylated polyclonal anti-MCMV antibodies (pα-MCMV Abs)

Anti-MCMV Smith hyperimmune sera were prepared as described before by Woolf et al. with slight modification [25]. Briefly, MCMV Smith was grown in MWFc, the virus was released by sonication and virus suspension was clarified to remove cellular debris by centrifugation (4000 *g* for 20 min). Mice were inoculated with 10^6^ TCID_50_ of clarified MCMV Smith intraperitoneally (IP), followed by two further IP inoculations at 2-week intervals. Afterwards, the plasma was collected at 7 days post last injection. IgG was isolated from plasma using Protein G Sepharose™ 4 Fast Flow (GE Healthcare), and protein concentration was determined by NanoDrop 2000 (Thermo Fisher Scientific). The purified antibodies were biotinylated with biotin reagents (EZ-Link® Sulfo-NHS-LC-Biotin, Thermo Fisher Scientific).

*pα-MCMV Abs* were tested for their reactivity against viral immediate early proteins, early proteins or late proteins by a co-localization assay of *pα-MCMV Abs* and murine monoclonal antibodies against immediate early protein (mouse anti-m123/IE1, CROMA101, isotype IgG1 (Capri, Croatia)), early protein (mouse anti-M112-113/E1, isotype IgG1 (Capri, Croatia)) and late protein (mouse anti-M55/gB, isotype IgG2b (Capri, Croatia)). The co-localization assay showed that *pα-MCMV Abs* recognized the viral early and late proteins but not viral immediate early proteins.

### Quantification of MCMV-infected cells in the nasal mucosa, lungs and submandibular glands

Immunofluorescence was used to quantify MCMV-infected cells in tissues (nasal mucosa, lungs and submandibular glands) that were MCMV HaNa1/MCMV Smith-positive after virus titration. The number of MCMV-infected cells in the nasal mucosa, submandibular glands and lungs of mice inoculated with the high dose (10^6^ TCID_50_/mouse) at 3, 7, 14 and 35 dpi was calculated. Forty consecutive cryosections (12 μm) per organ were fixed in 4% paraformaldehyde at 4 °C for 10 min and permeabilized with 0.1% Triton X-100 (Sigma) at room temperature (RT) for 10 min. Tissue sections were pretreated for 30 min with 10% negative goat serum and followed by incubating with *pα-MCMV Abs* (1:30) at 37 °C for 1 h. The cryosections were washed three times with PBS and incubated with the secondary antibodies: streptavidin Alexa-fluor® 488 conjugate, 1:200 (Invitrogen) at 37 °C for 1 h. After three washings, cell nuclei were stained with 10 μg/mL Hoechst 33342 (Invitrogen) at RT for 10 min. Finally, cryosections were mounted with glycerin-DABCO (Acros Organics).

Infected cells within each cryosection were quantified with the Leica TCS SPE laser-scanning confocal microscopy (magnification 200×, Leica Microsystems, GmbH, Wetzlar, Germany) according to the quantification method of Beyer et al. [26]. Forty consecutive 12 μm-sections were analyzed per organ. The total size of the analyzed sections was the sum of the sizes of the individual visual fields at a 200× magnification (diameter 1 mm). Finally, the number of MCMV-infected cells was calculated as a value per 10 mm^2^, independent of their localization and distribution within the cryosection.

### Identification of MCMV-infected cells in the nasal mucosa, lungs and submandibular glands

Immunofluorescence double staining was performed to identify MCMV-infected cells in the nasal mucosa, lungs and submandibular glands. Twelve μm viral antigen-positive cryosections of nasal mucosa, lungs and submandibular glands from 3, 7, 14 and 35 dpi were prepared following the aforementioned protocol. Sections were incubated at 37 °C for 1 h with *pα-MCMV Abs* (1:30) and cell markers (rabbit polyclonal anti-cytokeratin-18 for epithelia, 1:150 (Abcam); goat anti-olfactory marker protein for olfactory neurons [27], 1:500 (Wako); rat anti-mouse CD68/FITC-rat-anti-mouse F4/80 for tissue macrophages, 1:50 (AbD Serotec); hamster anti-mouse CD11c for dendritic cells, 1:50 (eBioscience); rat anti-mouse B220 for pan-B cells, 1:50 (Biolegend) and Alexa Fluor® 488-hamster-anti-mouse CD3 for T cells). The cryosections were washed three times with PBS and incubated at 37 °C for 1 h with the corresponding secondary antibodies: streptavidin-Texas Red-X or FITC conjugate, 1:200 (Invitrogen); FITC-goat-anti-rabbit IgG, 1:200 (Invitrogen); Alexa Fluor® 594-rabbit-anti-goat IgG, 1:200 (Invitrogen); Alexa Fluor® 488-goat-anti-rat IgG, 1:200 (Invitrogen); Alexa Fluor® 488-goat-anti-hamster IgG, 1:200 (Jackson ImmunoResearch) and Alexa Fluor® 488-goat-anti-rat IgG, 1:200 (Invitrogen). After washing three times, cell nuclei were counterstained with 10 μg/mL Hoechst 33342 (Invitrogen) at RT for 10 min. Finally, cryosections were mounted with glycerin-DABCO (Acros Organics) and analyzed with the Leica TCS SPE laser-scanning confocal microscopy (Leica Microsystems, GmbH, Wetzlar, Germany).

### Determination of total and isotype-specific anti-MCMV antibody titers

The titer of total and isotype specific anti-MCMV antibodies were determined in immunoperoxidase monolayer assays (IPMA) [28]. Briefly, monolayers of immortalized mouse embryo fibroblasts (MEFs) [29] in 96-well plates were inoculated with MCMV HaNa1 or MCMV Smith (10^3^ TCID_50_ per well), and cultivated for 3 days (37 °C, 5% CO_2_). Afterwards, the culture medium was removed, and cells were washed with PBS and dried at 37 °C for 1 h. The plates were covered with plastic covers and stored at −20 °C until use. Plates were thawed at RT and cells were fixed with 4% paraformaldehyde for 10 min at RT. The paraformaldehyde was removed, and cells were washed twice with PBS. Afterwards, the cells were treated with 100% methanol supplemented with 1% H_2_O_2_ at RT for 5 min. Plates were washed twice with PBS and serial twofold dilutions of plasma were added and incubated at 37 °C for 1 h. Plates were washed three times with PBS. To determine the virus-specific immunoglobulin classes and subclasses, 50 μL biotinylated secondary antibody (rat anti-mouse IgA biotin, 1:100, (eBioscience); rat anti-mouse IgM biotin, 1:100, (eBioscience); sheep anti-mouse IgG biotin, 1:100, (GE healthcare); rat anti-mouse IgG_1_ biotin, 1:100, (eBioscience); rat anti-mouse IgG_2a_ biotin, 1:100, (eBioscience); rat anti-mouse IgG_2b_ biotin, 1:100, (Biolegend); goat anti-mouse IgG_2c_ biotin, 1:100, (abcam); rat anti-mouse IgG_3_ biotin, 1:100, (Biolegend)) were added respectively and incubated at 37 °C for 1 h. Afterwards, plates were washed three times and 50 μl streptavidin-biotin horseradish peroxidase complex (1:200) was added per well and incubated at 37 °C for 1 h. Plates were washed three times and 50 μL of a substrate solution of 3-amino-9-ethylcarbazole (1/20) in 0.05 M acetate buffer, pH 5, with 0.024% H_2_O_2_ was added to each well and kept in RT for 30 min. Finally, the reaction was stopped with sodium acetate and the IPMA titer was calculated as the reciprocal value of the highest serum dilution that induced visual staining of infected MEFs as determined by a light microscopy (Olympus Optical Co., Hamburg, Germany). All aforementioned biotinylated secondary antibodies have been validated with sera from mice inoculated with influenza A/New Caledonia/20/99 (NC) virus by IPMA. In addition, the specificity of biotinylated secondary IgG antibodies had also been assessed with a panel of murine anti-MCMV Smith specific IgG monoclonal antibodies with known subclasses (mouse anti-m112-113, CROMA 103, IgG1; mouse anti-M123, IE1.01, IgG2a; mouse anti-m55-MCMV, CROMA7, IgG2b; mouse anti-m04-MCMV, m04-KAC.10, IgG2c; all were purchased from Capri, Croatia). No apparent cross reaction was found (data not shown).

### Complement-dependent neutralization test

Hyperimmune sera to CMVs were found to mainly contain complement-requiring neutralizing antibodies. Assays to measure neutralizing antibody titers were performed as described before by Farrell and Shellam and Lawson et al. with a few modifications [30,31]. At day one, serial twofold dilutions of heat-inactivated (56 °C, 30 min) plasma in MEM (from 1:2 to 1:512) were incubated with an equal volume containing 500 TCID_50_ MCMV Smith or 700 TCID_50_ MCMV HaNa1 for 23 h at 37 °C. Meanwhile, MWFc were trypsinized and seeded in 96-well plates with 100 μL per well at a concentration of 2.5 × 10^5^ cells/mL. After 23 h, 25 μL of guinea pig complement (0.5 μg/μL) was added to the virus/serum dilution mixtures for incubating another 1 h at 37 °C in a 5% CO_2_ incubator. Then, the medium of cells was removed and the mixtures of serum/virus/complement were added to the confluent cell monolayers. The plates were kept for 7 days at 37 °C. The neutralizing antibody titer was expressed as the reciprocal of the highest dilution that was able to completely block MCMV infection in MWFc.

## Results

### Growth kinetics of MCMV strains in MWFc

**Figure 1:**
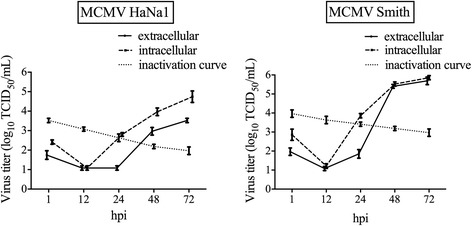
**Growth kinetics of MCMV HaNa1 and MCMV Smith in MWFc.** The virus titers generated in MWFc were determined, and growth curves of HaNa1 and Smith were plotted. The inactivation curve shows the drop of virus titers at 37 °C in culture medium due to inactivation events. The mean virus titer (log_10_ TCID_50_/mL) and standard deviation (*n* = 3) were shown in the diagram.

In the first experiment, the in vitro viral growth characteristics of MCMV HaNa1 and MCMV Smith were compared in MWFc, as shown in Figure 1. The study demonstrated that HaNa1 grew to a ~10-fold lower yield in comparison with the Smith strain and that HaNa1 isolate was more cell-associated than the Smith strain. Therefore, it can be stated that the Smith strain replicated in MWFc much more easily than the HaNa1 isolate.

### Virus titers in tissues

#### Low dose

**Figure 2:**
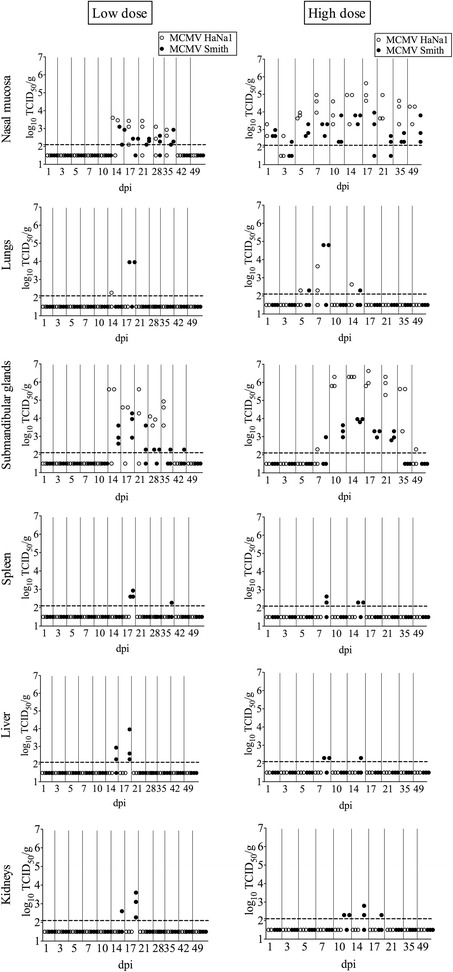
**Virus titers in the nasal mucosa, lungs, submandibular glands, saliva, plasma, spleen, liver and kidneys.** These tissues were collected from mice upon oronasal inoculation with either a low (10^4^TCID_50_/mouse, left column) or a high (10^6^TCID_50_/mouse, right column) inoculation dose. Virus titers (log_10_ TCID_50_/g or log_10_ TCID_50_/mL) were measured by titration. Open circles represent HaNa1-infected mice and closed circles represent Smith-infected mice. The detection limit for the titration (10^2.1^ TCID_50_/g) is shown by the horizontal dotted line.

After oronasal inoculation of 10^4^ TCID_50_ per mouse, MCMV HaNa1 was detected in the nasal mucosa from 14 till 35 dpi with the highest mean virus titer of 10^3.53^ TCID_50_/g at 14 dpi, in submandibular glands from 14 till 35 dpi with the highest mean virus titer of 10^4.93^ TCID_50_/g at 21 dpi (Figure 2), and in lungs, plasma, and saliva only at one time point (14 (*n* = 1), 21 (*n* = 2) and 28 (*n* = 1) dpi, respectively). The other organs (olfactory bulb, brain, pharynx, trachea, esophagus, small intestines, liver, kidneys, uterus, ovaries, thymus and spleen) remained all negative (under the detection limit).

MCMV Smith was detected in the nasal mucosa from 14 till 35 dpi and in submandibular glands from 14 till 42 dpi with the highest mean virus titer in the nasal mucosa (10^3.01^ TCID_50_/g) at 14 dpi and in the submandibular glands (10^3.72^ TCID_50_/g) at 17 dpi (Figure 2). Lungs were positive only at 17 dpi (*n* = 2). Saliva and plasma were negative during the course of infection. MCMV Smith led to a productive infection with virus replication in the spleen at 17 dpi (*n* = 3) and 35 dpi (*n* = 1), in the liver at 14 dpi (*n* = 2) and 17 dpi (*n* = 3), and in the kidneys at 14 dpi (*n* = 1) and 17 dpi (*n* = 3) (Figure 2). The other organs (olfactory bulb, brain, pharynx, trachea, esophagus, small intestines, uterus, ovaries and thymus) remained all negative (under the detection limit).

#### High dose

After oronasal inoculation of 10^6^ TCID_50_ per mouse, MCMV HaNa1 was detected in the nasal mucosa from 1 dpi till the end of the experiment (49 dpi) with the highest mean virus titer of 10^5.07^ TCID_50_/g at 17 dpi, in submandibular glands from 7 dpi till the end of the experiment 49 dpi with the highest mean virus titer of 10^6.3^ TCID_50_/g at 14 dpi, and in lungs at 5, 7 and 14 dpi with a low level of virus replication (Figure 2). At none of the collected time points post inoculation, infectious virus was detected in saliva and plasma. The other organs (olfactory bulb, brain, pharynx, trachea, esophagus, small intestines, liver, kidneys, uterus, ovaries, thymus and spleen) remained negative throughout the experiment (under the detection limit).

MCMV Smith was detected in the nasal mucosa from 1 dpi till the end of the experiment (49 dpi) with the highest mean virus titer of 10^3.63^ TCID_50_/g at 14 dpi, in submandibular glands from 7 dpi till 35 dpi with the highest mean virus titer of 10^3.91^ TCID_50_/g at 14 dpi, and in lungs from 5 dpi till 14 dpi except 10 dpi with a low level of virus replication close to the virus detection limit except at 7 dpi with a virus titer of 10^4.8^ TCID_50_/g in 2 out of 3 mice (Figure 2). MCMV Smith led to a productive infection with virus replication in the spleen at 7 dpi (*n* = 2) and 14 dpi (*n* = 2), in the liver at 7 dpi (*n* = 2) and 14 dpi (*n* = 1), and in the kidneys at 10 dpi (*n* = 2), 14 dpi (*n* = 2) and 17 dpi (*n* = 1) (Figure 2). Infectious virus was not detected in saliva and plasma at any indicated time point. No virus was detected in the control mice. The other organs (olfactory bulb, brain, pharynx, trachea, esophagus, small intestines, uterus, ovaries and thymus) remained negative throughout the experiment (under the detection limit).

### Co-culture of PBMC with MWFc

At none of the collected time points post inoculation, cell-associated virus was detected by co-culture for both strains at a low inoculation dose. At a high inoculation dose, cell-associated virus was detected in PBMC for both strains: at 7 dpi (*n* = 2) and 10 dpi (*n* = 1) in HaNa1-infected mice; at 7 dpi (*n* = 2) in Smith-infected mice.

### Quantification of MCMV-infected cells in the nasal mucosa, lungs and submandibular glands

**Figure 3:**
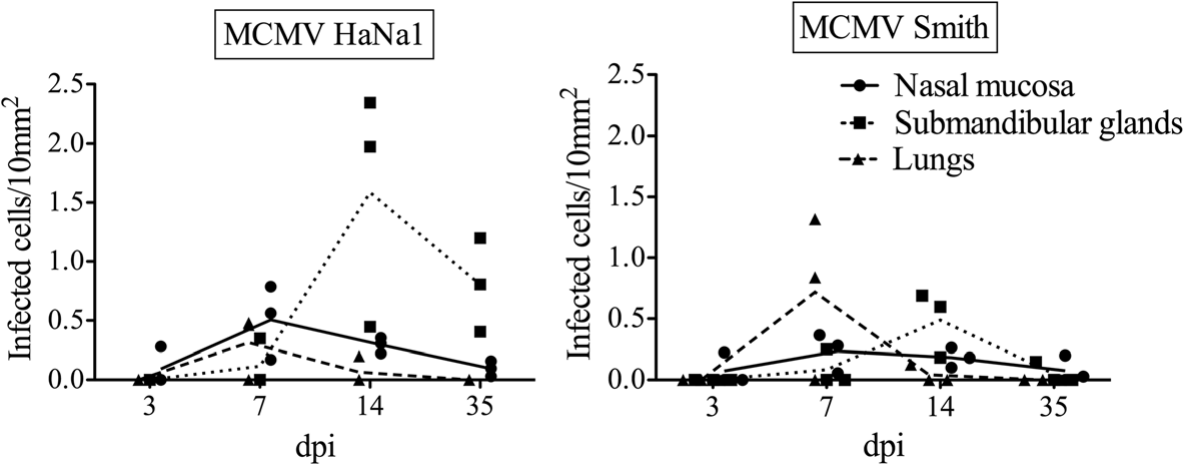
**Quantification of MCMV-infected cells in the nasal mucosa, submandibular glands and lungs.** Each time point has three individual animals. Forty consecutive cryosections per tissue were evaluated by immunofluorescence microscopy. The number of infected cells per 10 mm^2^ is shown. The average values of infected cells at different time points were connected by lines.

The nasal mucosa, lungs and submandibular glands from mice that received a high inoculation dose were collected at 3, 7, 14 and 35 dpi and the number of MCMV-infected cells in these organs was quantified. The same samples from mock-inoculated mice were negative for MCMV. Figure 3 shows the number of MCMV-infected cells in these organs.

For HaNa1-infected mice, infected cells in the nasal mucosa were first detected at 3 dpi (0.09 cells per 10 mm^2^), peaked at 7 dpi (0.51 cells per 10 mm^2^) and dropped sharply afterwards (at 35 dpi: 0.09 cells per 10 mm^2^). In lungs, they were first detected and also peaked at 7 dpi (0.47 cells per 10 mm^2^), decreased afterwards significantly and were undetectable at 35 dpi. In submandibular glands, they were first noticed at 7 dpi (0.13 cells per 10 mm^2^), peaked at 14 dpi (1.59 cells per 10 mm^2^), and dropped sharply afterwards (0.80 cells per 10 mm^2^ at 35 dpi).

For Smith-infected mice, MCMV-infected cells in the nasal mucosa were first detected at 3 dpi (0.07 cells per 10 mm^2^), peaked at 7 dpi (0.23 cells per 10 mm^2^) and then decreased till 35 dpi (0.08 cells per 10 mm^2^). In lungs, they were first detected and also peaked at 7 dpi (0.72 cells per 10 mm^2^), afterwards fell dramatically and were undetectable at 35 dpi. In submandibular glands, they were first detected at 7 dpi (0.08 cells per 10 mm^2^), reached a peak at 14 dpi (0.49 cells per 10 mm^2^) and decreased afterwards (0.05 cells per 10 mm^2^ at 35 dpi).

### Identification of MCMV-infected cells in the nasal mucosa, lungs and submandibular glands

**Figure 4:**
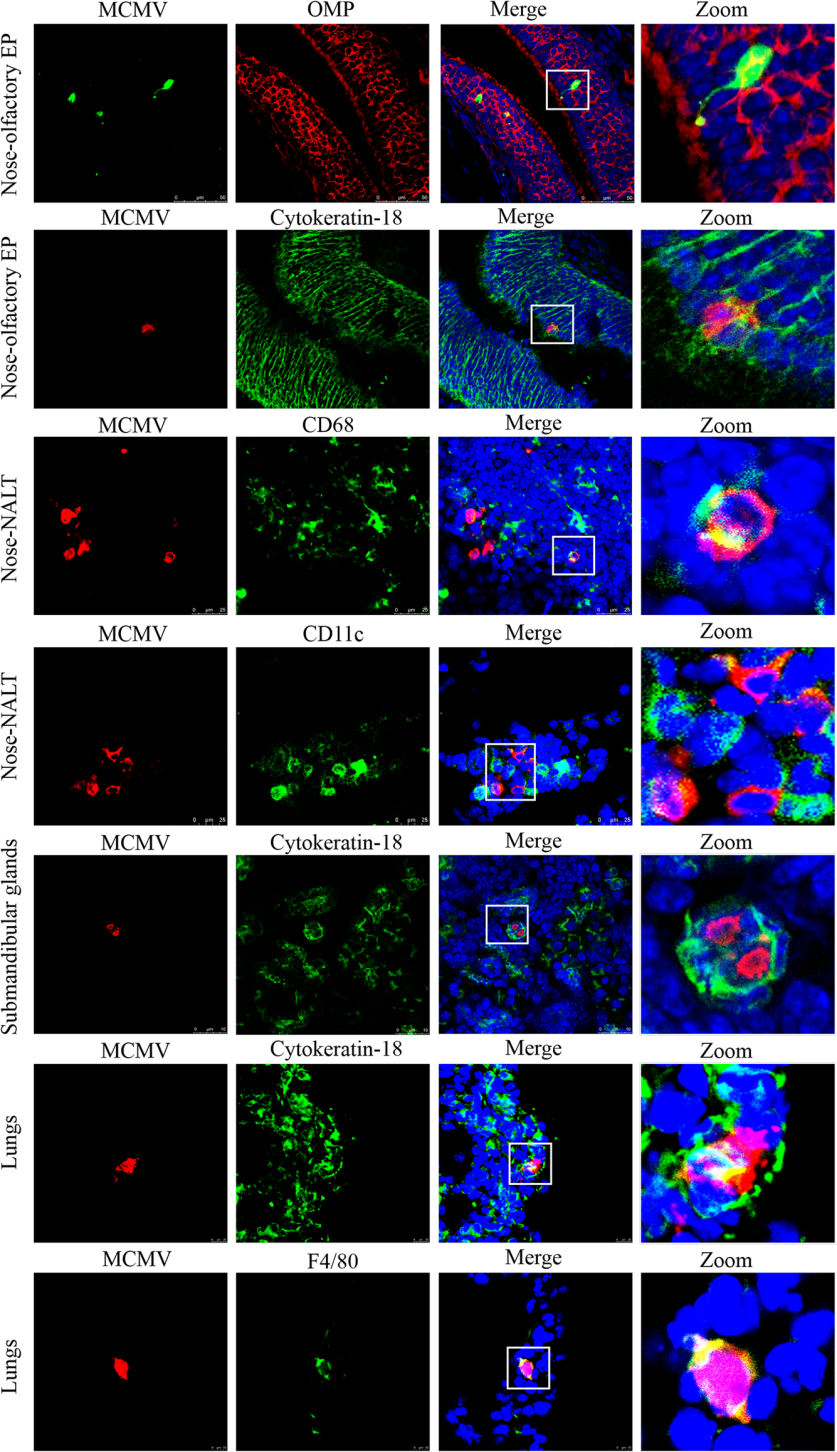
**Identification of MCMV-infected cells in the nasal mucosa, lungs and submandibular glands.** Nuclei were counterstained with Hoechst (Blue). Co-localization appears yellow in the merged images. The zoomed images show the boxed region of merged layers. Cryosections of the nasal mucosa were double-stained with antibodies against MCMV antigens (by *pα-MCMV Abs*) and cell markers: OMP (olfactory neuron marker), cytokeratin-18 (epithelial marker), CD68 (tissue macrophages) and CD11c (dendritic cells). Olfactory neurons and sustentacular cells of the olfactory epithelium (olfactory EP) in the nasal mucosa were susceptible for MCMV. Infected cells of NALT in the nasal mucosa were CD68^+^/CD11c^+^. Epithelial cells (cytokeratin-18^+^) of submandibular glands were susceptible for MCMV. Epithelial cells (cytokeratin-18^+^) and macrophages (F4/80^+^) were positive for MCMV in lungs.

Identification of the MCMV-infected cells in target tissues would help us to understand the cell tropism of MCMV. The nasal mucosa, lungs and submandibular glands from mice inoculated with a high dose were collected at 3, 7, 14 and 35 dpi to be stained for MCMV antigens and cellular markers simultaneously. The morphology of MCMV-positive cells consisted of a big round or oval unstained nucleus surrounded by a thick rim of positive cytoplasm (Figure 4). Since both strains gave similar results, only staining of cryosections from HaNa1-infected mice were presented here. In the nasal mucosa, the infected cells were only found in the olfactory epithelium and nasopharynx associated lymph tissue (NALT) from 3 dpi onwards. Based on our results, viral proteins were expressed in both sustentacular cells and neurons in the olfactory epithelium, and in CD68/CD11c positive cells (macrophages/dendritic cells) but not in B220/CD3 positive cells (B cells/T cells) in the NALT. In lungs, epithelial cells and macrophages were susceptible cell types at 7 and 14 dpi. In submandibular glands, only epithelial cells were susceptible at 7, 14 and 35 dpi.

### Serology

#### Viral-specific antibodies by IPMA

**Figure 5:**
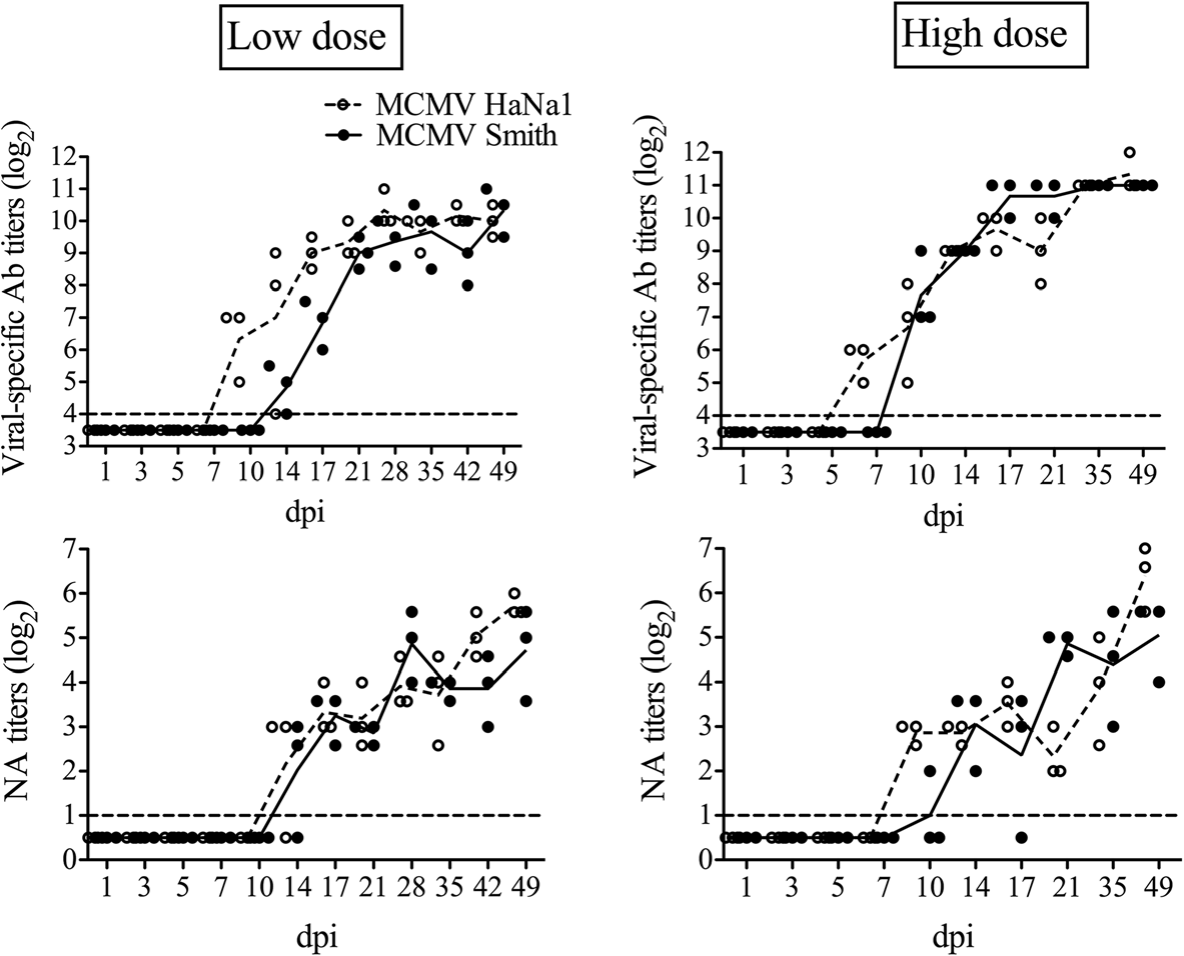
**Viral-specific antibody titers by IPMA and neutralizing antibody (NA) titers by NA.** They were determined in the Smith or HaNa1-infected mice that had been inoculated with either a low (10^4^TCID_50_/mouse) or a high (10^6^TCID_50_/mouse) inoculation dose. Each time point represents the mean of 3 BALB/c mice ± standard deviation. The detection limit was shown by the dotted line.

Low dose-MCMV HaNa1-specific antibodies were first detected at 10 dpi. Afterwards, titers rose and reached a maximal level at 21 dpi; MCMV Smith-specific antibodies showed a similar course with the exception that they appeared later (at 14 dpi) (Figure 5). High dose-The high dose (10^6^ TCID_50_/mouse) reduced the time of appearance of antibodies (HaNa1 at 7 dpi; Smith at 10 dpi). Both reached a maximal level at 17 dpi.

#### Subclasses determination by IPMA

Low dose-The results of specific Ig classes and IgG subclasses determination showed that only IgG2a was detectable throughout the whole experiment, whereas the other (IgA, IgM and other IgG subclasses (IgG1, IgG2b, IgG2c and IgG3)) were not (Table 1). IgG_2a_ subclass was detected earlier in HaNa1-infected mice at 14 dpi than in Smith-infected mice at 17 dpi. High dose-IgG2a was the main antibody subclass (Table 2). IgG1 and IgG2c antibodies were also detected for both strains but with lower titers (lower than or equal to 64) albeit at 35 and 49 dpi.

**Table 1:** **Course of isotype-specific anti-MCMV HaNa1 or anti-MCMV Smith antibody titers in BALB/c mice inoculated with 10** ^**4**^ **TCID**_**50**_ **per mouse**

**Strains**	**Classes/subclasses**	**Antibody titers**											
		**1d**	**3d**	**5d**	**7d**	**10d**	**14d**	**17d**	**21d**	**28d**	**35d**	**42d**	**49d**
MCMV HaNa1	IgA	−/−/-	−/−/-	−/−/-	−/−/-	−/−/-	−/−/-	−/−/-	−/−/-	−/−/-	−/−/-	−/−/-	−/−/-
	IgM	−/−/-	−/−/-	−/−/-	−/−/-	−/−/-	−/−/-	−/−/-	−/−/-	−/−/-	−/−/-	−/−/-	−/−/-
	IgG_1_	−/−/-	−/−/-	−/−/-	−/−/-	−/−/-	−/−/-	−/−/-	−/−/-	−/−/-	−/−/-	−/−/-	−/−/-
	IgG_2a_	−/−/-	−/−/-	−/−/-	−/−/-	−/−/-	32/−/−	32/32/16	64/32/64	128/256/128	128/64/128	128/128/128	128/128/128
	IgG_2b_	−/−/-	−/−/-	−/−/-	−/−/-	−/−/-	−/−/-	−/−/-	−/−/-	−/−/-	−/−/-	−/−/-	−/−/-
	IgG_2c_	−/−/-	−/−/-	−/−/-	−/−/-	−/−/-	−/−/-	−/−/-	−/−/-	−/−/-	−/−/-	−/−/-	−/−/-
	IgG_3_	−/−/-	−/−/-	−/−/-	−/−/-	−/−/-	−/−/-	−/−/-	−/−/-	−/−/-	−/−/-	−/−/-	−/−/-
MCMV Smith	IgA	−/−/-	−/−/-	−/−/-	−/−/-	−/−/-	−/−/-	−/−/-	−/−/-	−/−/-	−/−/-	−/−/-	−/−/-
	IgM	−/−/-	−/−/-	−/−/-	−/−/-	−/−/-	−/−/-	−/−/-	−/−/-	−/−/-	−/−/-	−/−/-	−/−/-
	IgG_1_	−/−/-	−/−/-	−/−/-	−/−/-	−/−/-	−/−/-	−/−/-	−/−/-	−/−/-	−/−/-	−/−/-	−/−/-
	IgG_2a_	−/−/-	−/−/-	−/−/-	−/−/-	−/−/-	−/−/-	32/32/32	32/64/64	64/128/32	128/64/128	128/64/128	128/128/128
	IgG_2b_	−/−/-	−/−/-	−/−/-	−/−/-	−/−/-	−/−/-	−/−/-	−/−/-	−/−/-	−/−/-	−/−/-	−/−/-
	IgG_2c_	−/−/-	−/−/-	−/−/-	−/−/-	−/−/-	−/−/-	−/−/-	−/−/-	−/−/-	−/−/-	−/−/-	−/−/-
	IgG_3_	−/−/-	−/−/-	−/−/-	−/−/-	−/−/-	−/−/-	−/−/-	−/−/-	−/−/-	−/−/-	−/−/-	−/−/-

“-” represents that the antibody titer was under the detection limit (1/16).

**Table 2:** **Course of isotype-specific anti-MCMV HaNa1 or anti-MCMV Smith antibody titers in BALB/c mice inoculated with 10** ^**6**^ **TCID**_**50**_ **per mouse**

**Strains**	**Classes/subclasses**	**Antibody titers**									
		**1d**	**3d**	**5d**	**7d**	**10d**	**14d**	**17d**	**21d**	**35d**	**49d**
MCMV HaNa1	IgA	−/−/-	−/−/-	−/−/-	−/−/-	−/−/-	−/−/-	−/−/-	−/−/-	−/−/-	−/−/-
	IgM	−/−/-	−/−/-	−/−/-	−/−/-	−/−/-	−/−/-	−/−/-	−/−/-	−/−/-	−/−/-
	IgG_1_	−/−/-	−/−/-	−/−/-	−/−/-	−/−/-	−/−/-	−/−/-	−/−/-	128/16/64	32/64/64
	IgG_2a_	−/−/-	−/−/-	−/−/-	−/−/-	64/16/32	256/128/256	256/256/512	128/256/256	1024/1024/1024	256/1024/1024
	IgG_2b_	−/−/-	−/−/-	−/−/-	−/−/-	−/−/-	−/−/-	−/−/-	−/−/-	−/−/-	−/−/-
	IgG_2c_	−/−/-	−/−/-	−/−/-	−/−/-	−/−/-	−/−/-	−/−/-	−/−/-	−/−/-	64/32/64
	IgG_3_	−/−/-	−/−/-	−/−/-	−/−/-	−/−/-	−/−/-	−/−/-	−/−/-	−/−/-	−/−/-
MCMV Smith	IgA	−/−/-	−/−/-	−/−/-	−/−/-	−/−/-	−/−/-	−/−/-	−/−/-	−/−/-	−/−/-
	IgM	−/−/-	−/−/-	−/−/-	−/−/-	−/−/-	−/−/-	−/−/-	−/−/-	−/−/-	−/−/-
	IgG_1_	−/−/-	−/−/-	−/−/-	−/−/-	−/−/-	−/−/-	−/−/-	−/−/-	-/32/-	64/32/-
	IgG_2a_	−/−/-	−/−/-	−/−/-	−/−/-	32/-/64	64/128/128	256/128/256	512/512/256	512/512/1024	1024/512/512
	IgG_2b_	−/−/-	−/−/-	−/−/-	−/−/-	−/−/-	−/−/-	−/−/-	−/−/-	−/−/-	−/−/-
	IgG_2c_	−/−/-	−/−/-	−/−/-	−/−/-	−/−/-	−/−/-	−/−/-	−/−/-	16/32/16	64/16/64
	IgG_3_	−/−/-	−/−/-	−/−/-	−/−/-	−/−/-	−/−/-	−/−/-	−/−/-	−/−/-	−/−/-

“-” represents that the antibody titer was under the detection limit (1/16).

#### Complement-dependent neutralizing antibodies by NA test

Neutralizing antibodies without adding guinea pig complement were only detected at 35–49 dpi with low NA titers (lower than or equal to 2) for both strains (data not shown). Complement-dependent neutralizing antibodies from mice inoculated with a low inoculation dose were first detected at 14 dpi, afterwards increased till the end of experiment at 49 dpi (Figure 5). A similar pattern was found with a high inoculation dose, except that neutralizing antibodies appeared earlier at 10 dpi.

## Discussion

To date, MCMV has been widely used in laboratory models for studying HCMV infections. Almost all the laboratory research on MCMV used either the Smith or the Smith-derived K181 strains, which are the two standard laboratory MCMV stains [17]. These strains have been serially passaged in vitro or in vivo for more than 60 years, which likely caused genetic and biological changes. Furthermore, non-natural inoculation routes (subcutaneously, intraperitoneally, or intravenously) have been utilized to inoculate animals in the previous studies, thus bypassing the mucosal sites of virus replication and the local immune response [4,13]. Therefore, there is limited information on natural infections with low passaged MCMV. Here, an MCMV infection model has been established that mimics natural infection using a recently Belgian MCMV isolate HaNa1. We found that upon oronasal inoculation: 1) the nasal mucosa and submandibular glands were the main sites of productive MCMV replication; 2) the Smith strain established a productive infection in spleen, liver and kidneys, whereas the HaNa1 isolate did not; 3) increasing the inoculation dose strongly elevated virus production in the nasal mucosa and submandibular glands, and also reduced the time of appearance of antibodies.

In the present study, it was examined whether HaNa1 and Smith differed in viral growth in vitro and in vivo. The growth kinetics in vitro demonstrated that the Smith strain replicated to a ~10-fold higher yield than the HaNa1 isolate. In contrast to the in vitro situation, in the nasal mucosa and submandibular glands, HaNa1 replicated to higher titers than Smith in vivo. This is consistent with their passage history. The MCMV with more passages in cells grows better in vitro but loses part of its replication ability at the entry and exit sites in vivo [32,33].

In vivo, both stains (Smith and HaNa1) were first detected in the nasal mucosa. Increasing the inoculation dose elevated virus production leading to early detection and higher virus titers. HaNa1 reached higher virus titers than Smith. In our study, the nasal mucosa was shown for the first time to be a susceptible organ for MCMV. Based on similar characteristics of human and murine CMVs, we hypothesize that the nasal mucosa might also be a target organ for HCMV. In line with this, there have been several reports on the detection of HCMV in nasopharyngeal carcinomas, sinusitis and nasal polyposis [34-37]. In lungs, both strains showed a very restricted replication during the first three weeks after post inoculation, after which the infection was controlled. Our finding is consistent with a previous study [38]. The submandibular gland is another target organ for both strains. In contrast with the viral replication in the nasal mucosa, the viral replication in the submandibular glands always started after one week post inoculation and lasted longer than 49 dpi post inoculation. HaNa1 reached much higher virus titers (>100 fold) than Smith. Similarly to the nasal mucosa, increasing the inoculation dose enhanced virus production in the submandibular glands. CMVs have been reported to mainly use salivary glands as target organ for virus persistence and shedding into saliva [13,39,40]. However, in our study, HaNa1 was detected in saliva only at one time point within one mouse. The low level of virus titers in saliva was quite surprising, as CMVs are thought to be transmitted orally via saliva. In future studies, these conflicting data will be further examined.

Cell-associated virus in PBMC was detected at 7–10 dpi for both strains with a high inoculation dose. This shows that circulating PBMC are involved in the dissemination of MCMV, which corresponds with a previous study [41]. In our study, only the Smith strain was detectable by virus titration in spleen, liver and kidneys from the second week post inoculation onwards, providing evidence that only the Smith strain can establish a productive infection in internal organs of adult mice, whereas HaNa1 cannot. The latter is similar to the outcome of an HCMV primary infection in immunocompetent adults, during which it is only causing a limited virus-associated spread to the salivary glands but not to multiple internal organs [42].

Quantification of MCMV-infected cells in the nasal mucosa, lungs and submandibular glands revealed a good correlation between virus titers and infected cells. Identification of MCMV-infected cells demonstrated that in the nasal mucosa, olfactory neurons as well as sustentacular cells in the olfactory neuroepithelium and CD68/CD11c positive cells (macrophages/dendritic cells) in NALT were the main susceptible cell types. Targeting the olfactory neurons raised the question if CMV may damage the smell [43,44]. This will be investigated in the future. According to the staining results, CD68/CD11c positive cells (macrophages/dendritic cells) in NALT were infected from 3 dpi onwards, which indicates that NALT plays a very important role in MCMV infection. Therefore, we presume that the virus may be transmitted via lymphatic circulation to draining lymph nodes, ending up in the blood circulation. In lungs, both epithelial cells and macrophages are susceptible to both MCMV strains, which are the main cause of pneumonitis caused by MCMV [21,38]. It is also a frequently observed manifestation of HCMV infection [45,46]. In submandibular glands, epithelial cells are the main susceptible cell type for both MCMV strains, which is consistent with earlier published data [47]. However, up till now it is unclear how CMV reaches the submandibular glands and becomes transferred to the epithelial cells.

Serological analysis showed that IgG2a was the antibody subclass that was mainly produced except that low titers of IgG1 and IgG2c were also detected in mice inoculated with a high dose at 35–49 dpi. IgM was not detected through the whole course of the experiments for both MCMV strains. This could be due to the low sensitivity of the MCMV-specific IPMA, which is consistent with our positive control (low sensitivity of the mice adapted influenza-specific IPMA), or the suppressive effect of MCMV on the production of IgM by T cell cytokines. T cell cytokines are responsible for the immunoglobulin class switching mechanism in mouse and human [48]. The strong induction of IgG2a is generally known to be mediated by interferon γ (IFNγ) [49,50]. Because IFNγ as well as other T cell cytokines were not evaluated in this study, we could not assess the role of IFNγ in the orientation of the antibody isotype switch. The complement-dependent neutralization test demonstrated that complement plays a critical role in neutralizing MCMV since antibodies without complement/with inactivated complement did not neutralize MCMV infection. Since IgG2a is the predominant viral-specific antibody, we can state that complement-dependent IgG2a-mediated inactivation of MCMV is an important anti-MCMV defense. This is consistent with the characteristics of antibody isotype IgG2a to fix complement in mice [51]. Neutralizing antibody titers were higher at 35 and 49 dpi, which may explain in part the clearance of HaNa1 at 49 dpi and Smith at 35 and 49 dpi in submandibular glands in the high inoculation dose group. Similar patterns also occurred for both strains at 42 and 49 dpi in the low inoculation dose groups. The control of virus infection at the end of the experiments is also most probably mediated by the cell-mediated immunity, which is generally considered to be the most important factor in controlling CMV infections [52,53].

In summary, mouse models with MCMV that are mimicking natural infection were set up in the present study. By the use of two MCMV strains (Smith and HaNa1), we found that infections started in the upper respiratory tract, after which the virus spreads via a cell-associated viremia to other target organs such as spleen, liver, kidneys and submandibular glands. The Smith strain caused a productive infection in spleen, liver and kidneys, whereas the HaNa1 isolate did not. The latter is similar to the outcome of an HCMV primary infection in immunocompetent hosts; it is only causing a limited virus-associated spread to the salivary glands but not to multiple internal organs. Therefore, the newly isolated MCMV HaNa1 isolate is interesting to be used in mouse models in order to get better insights into HCMV natural infections in immunocompetent hosts via oronasal exposure. Increasing the inoculation dose strongly elevated virus production in the nasal mucosa and submandibular glands and cell-associated viremia during the early stage of infection, reduced the time of appearance of antibodies, and increased the level of antibodies. In this study, we predominantly focused on the kinetics of virus production in different organs. As known for a long time, CMVs spread in a strong cell-associated way. This was not investigated in depth in the present study. Therefore, in the near future, we will focus on the kinetics of MCMV infected leukocytes (cell-associated virus) in order to better understand the viral link between the respiratory tract, as portal of entry, and the submandibular glands, as portal of exit. It is very important to understand how MCMV starts up replication in the submandibular glands and maintains its replication in this organ for a long period of time.
